# Supplementary material for: Finger-inspired rigid-soft hybrid tactile sensor with superior sensitivity at high frequency
Source: Nat Commun. 2022 Aug 29;13:5076. doi: 10.1038/s41467-022-32827-7 (PMC9422944; doi:10.1038/s41467-022-32827-7)
Supplement: Supplementary file 1 — Supplementary Information [file 41467_2022_32827_MOESM1_ESM.pdf]

Supplementary Information

Finger-Inspired Rigid-Soft Hybrid Tactile Sensor with Superior Sensitivity at High Frequency

Supplementary Table 1 Comparison of the sensing performance between our RSHTS and existing piezoelectric tactile sensors.

Ref.	Main materials	Experimental maximum sensitivity	Theoretical limit sensitivity value	Force range	Bandwidth	Working mode	Force direction detection
1	Si/PVDF	7 V N^{-1}	/ [a]	0.008-1.35 N	/	d_{33}	Normal
2,3	PDMS/PVDF/PDMS	7.32 mV N^{-1} [2]	33 pC N^{-1} [2]	0.5-4 N [2]	1-100 Hz [2]	d_{33}	Normal
4	PDMS/ PVDF-ZnO nanofibers/PDMS	/	/ [a]	0.35-8.75 N	/	d_{33}	Normal
5,6	PDMS/ PVDF/PDMS	8.83 V N^{-1} [5]	/ [a] [5]	0.01-1 N [5]	1-50 Hz [5]	d_{33}	Normal
7	PDMS/PVDF/PDMS	6.62 pC N^{-1}	15±3 pC N^{-1}	0-1.5 N	5-400 Hz	d_{33}	Normal & shear
8	PVDF	12.6±0.8 mV N^{-1}	33 pC N^{-1}	0.08-0.28 N	1-10 Hz	d_{33}	Normal & shear
9	PVDF	/	10 pC N^{-1}	/	/	d_{33}	Normal & shear
10,11	PET/P(VDF-TrFE)/PI	~50 mV N^{-1} [10]	/	0.5-3.0 N [10]	10-200 Hz [10]	d_{33}	Normal
12,13	PI/P(VDF-TrFE)/PI	228.2 mV N^{-1} [12]	32.5 pC N^{-1} [12]	0.5-10 N [12]	/	d_{33}	Normal
14,15	PDMS/PVDF-MWCNT/PDMS	3.0 pC N^{-1} [14]	/	2-8 N [14]	5-15 Hz [14]	d_{33}	Normal & shear
16	PI/PVDF/ PI	0.21 mV N^{-1}	33 pC N^{-1}	0.7-10 N	1-120 Hz	d_{33}	Normal
17	PI/PVDF/ PI	430 mV N^{-1}	47 pC N^{-1}	~0.5-2 N	~200 Hz	d_{33}	Normal
**Our work**	PDMS/PVDF/Silicone	346.5 pC N^{-1}	21 pC N^{-1} (d_{33} mode)	0.009-4.3 N	5-600 Hz	d_{31}	Normal & shear

[a] The piezoelectric coefficient of the PVDF used in these reports is not given directly.

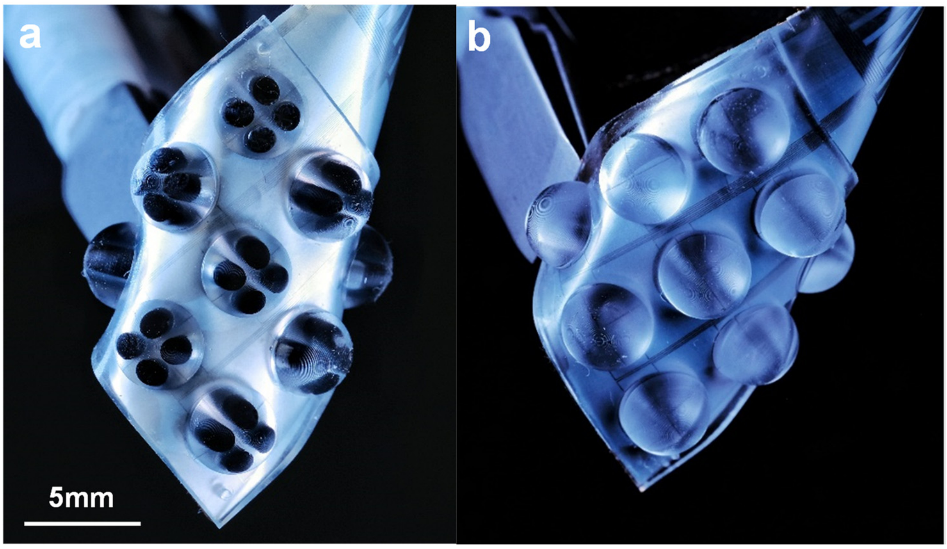

Supplementary Fig. 1 | Comparison of flexibility between the RSHTS and the control sensor without rigid pillars. a Photograph of a fabricated RSHTS array. **b** Photograph of a fabricated control sensor array without rigid pillars. From the photos, RSHTS shows similar flexibility to purely soft tactile sensor without rigid pillars.

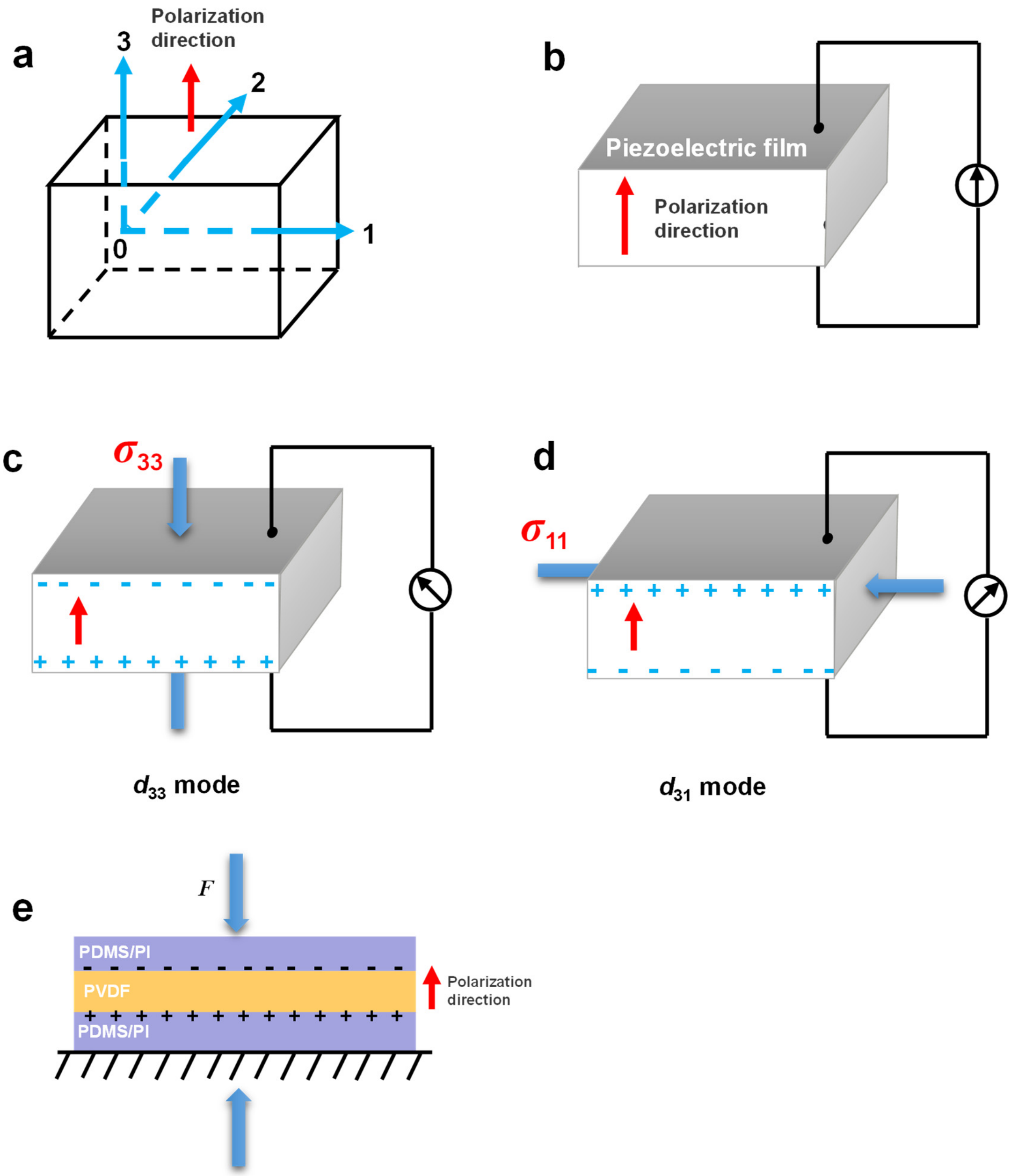

Supplementary Fig. 2 | Working mode of piezoelectric sensory layer. a Schematic of three-dimensional system of coordinates, where the polarization direction of piezoelectric material is along Direction 3. **b** Schematic of polarized piezoelectric film without an external load. **c** Schematic of d_{33} mode of piezoelectric film upon a load σ_{33}. **d** Schematic of d_{31} mode of piezoelectric film upon a load σ_{11}. **e** Schematic of traditional piezoelectric tactile sensors using d_{33} mode.

The piezoelectric sensor is an electronic device that converts mechanical stress into electric charge through the piezoelectric effect. As a mechanical force is applied to piezoelectric film, the generated electric displacement D_3 of PVDF (Supplementary Fig. 2b) without an external electric field can be expressed by:

$$D_3 = d_{31}\sigma_{11} + d_{32}\sigma_{22} + d_{33}\sigma_{33} \quad (1)$$

The generated charge Q of the piezoelectric film with an electrode area of A is

$$Q = (d_{31}\sigma_{11} + d_{32}\sigma_{22} + d_{33}\sigma_{33})A \tag{2}$$

where σ_{ij} (i, j = 1, 2, 3) is the mechanical stress, and d_{mn} (m = 1, 2, 3; n = 1, 2, 3, 4, 5, 6) is the piezoelectric constant in cartesian coordinate.

Two piezoelectric modes, d_{31} and d_{33} are commonly used in piezoelectric devices. The relative directions of the electric field and the stress distinguish them: d_{33} mode when the generated electric field is parallel to stress (Supplementary Fig. 2c), and d_{31} mode when generated electric field is perpendicular to stress (Supplementary Fig. 2d). Conventional piezoelectric tactile sensors are a d_{33} configuration in which a piezoelectric layer is sandwiched between top and bottom flat substrates (Supplementary Fig. 2e). For d_{33} mode (the in-plane normal stress σ_{11} is neglected compared to the out-plane normal stress σ_{33}), the generated charge Q_0 is

$$Q_0 \approx d_{33}\sigma_{33}A \tag{3}$$

Therefore, the sensitivity S_0 of the common piezoelectric tactile sensors working in d_{33} mode can be expressed as

$$S_0 = \frac{\Delta Q}{\Delta F} \approx \frac{d_{33}\sigma_{33}^{0}A}{F} = \frac{d_{33}kF}{F} = d_{33}k \leq d_{33}, (k \leq 1) \tag{4}$$

Different from the common piezoelectric tactile sensors, our RSHTS using a design of a rigid-soft hybrid force-transmission-layer in combination with a soft bottom substrate makes the sensory layer work in d_{31} mode, based on Eq. (2), its sensitivity S can be expressed as

$$S = \frac{\Delta Q}{\Delta F} = \frac{(d_{31}\sigma_{11} + d_{32}\sigma_{22} + d_{33}\sigma_{33})A}{F} \tag{5}$$

where σ_{22} is equal to σ_{11}, thus, S is rewritten as

$$S = \frac{\Delta Q}{\Delta F} = \frac{((d_{31} + d_{32})\sigma_{11} + d_{33}\sigma_{33})A}{F} \tag{6}$$

Thus,

$$\frac{S}{S_0} = \frac{(d_{31} + d_{32})\sigma_{11} + d_{33}\sigma_{33}}{d_{33}\sigma_{33}^{0}} \tag{7}$$

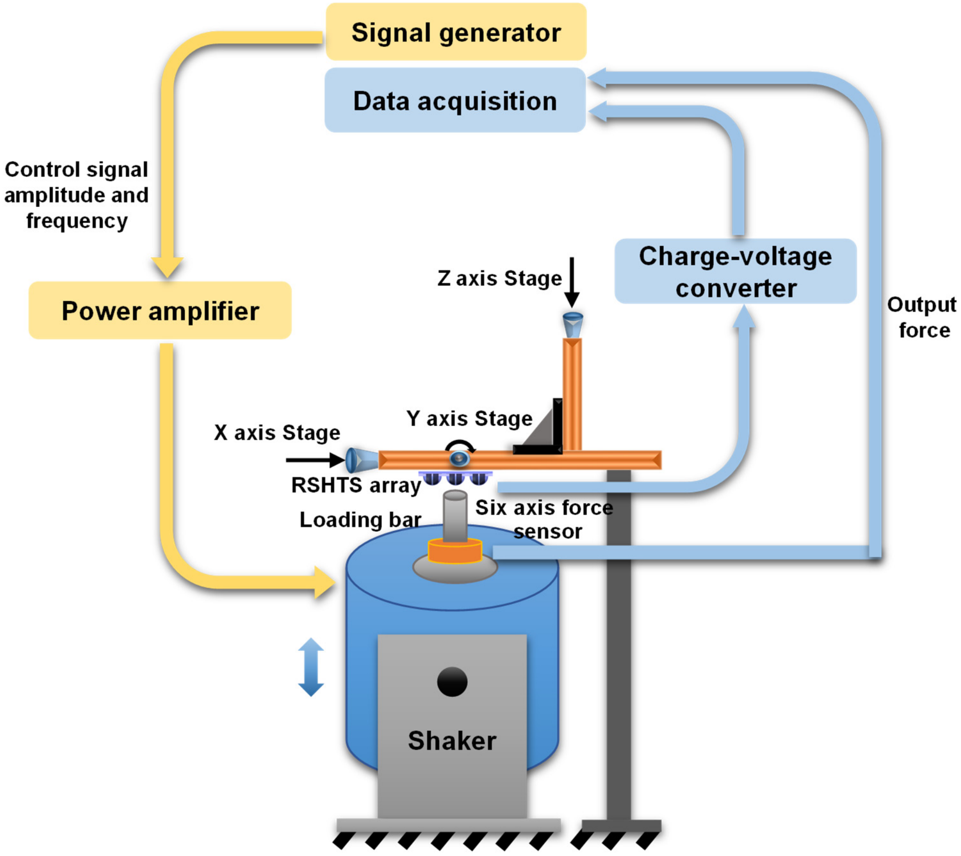

Supplementary Fig. 3 | Schematic of a vibration test system to provide diverse external stimuli for the tactile sensor. Details can be found in the *Measurements* section.

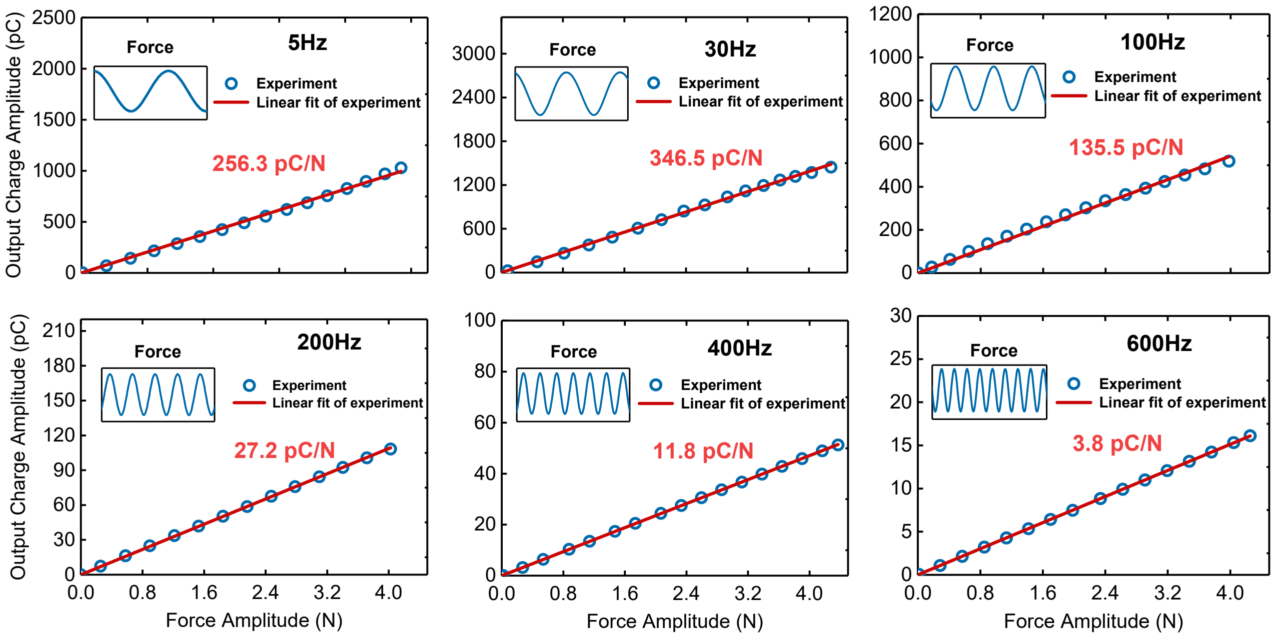

Supplementary Fig. 4 | Frequency response of the RSHTS. Output charge of the RSHTS as a function of applied normal force measured at 5–600 Hz.

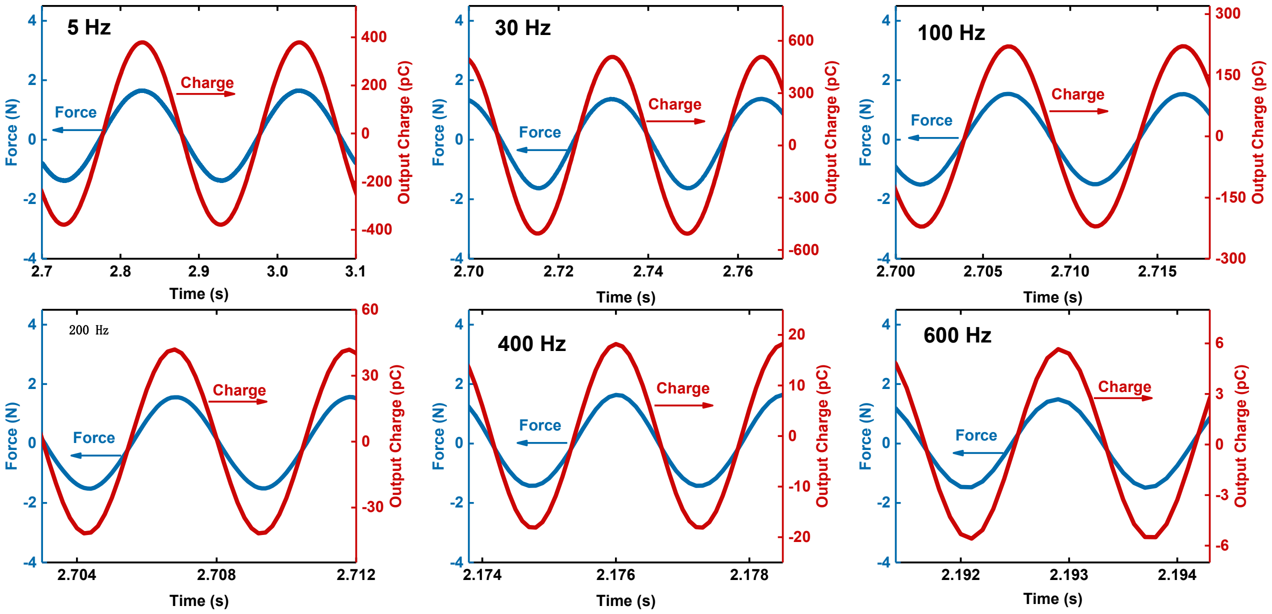

Supplementary Fig. 5 | Dynamic response of the commercial six-axis force sensor and the RSHTS under a sinusoidal force with an amplitude of 1.7 N at 5–600 Hz. The results suggest that the RSHTS has good frequency response.

Supplementary Table 2 Simulation parameters of tactile sensors in this work.

Parameters	Young's modulus	Poisson's ratio
**PDMS**	2.0 MPa	0.46
**Epoxy**	4.1 GPa	0.32
**Silicone**	1.0 MPa	0.4
**PVDF**	3.0 GPa	0.35
**Steel**	2.1 GPa	0.286

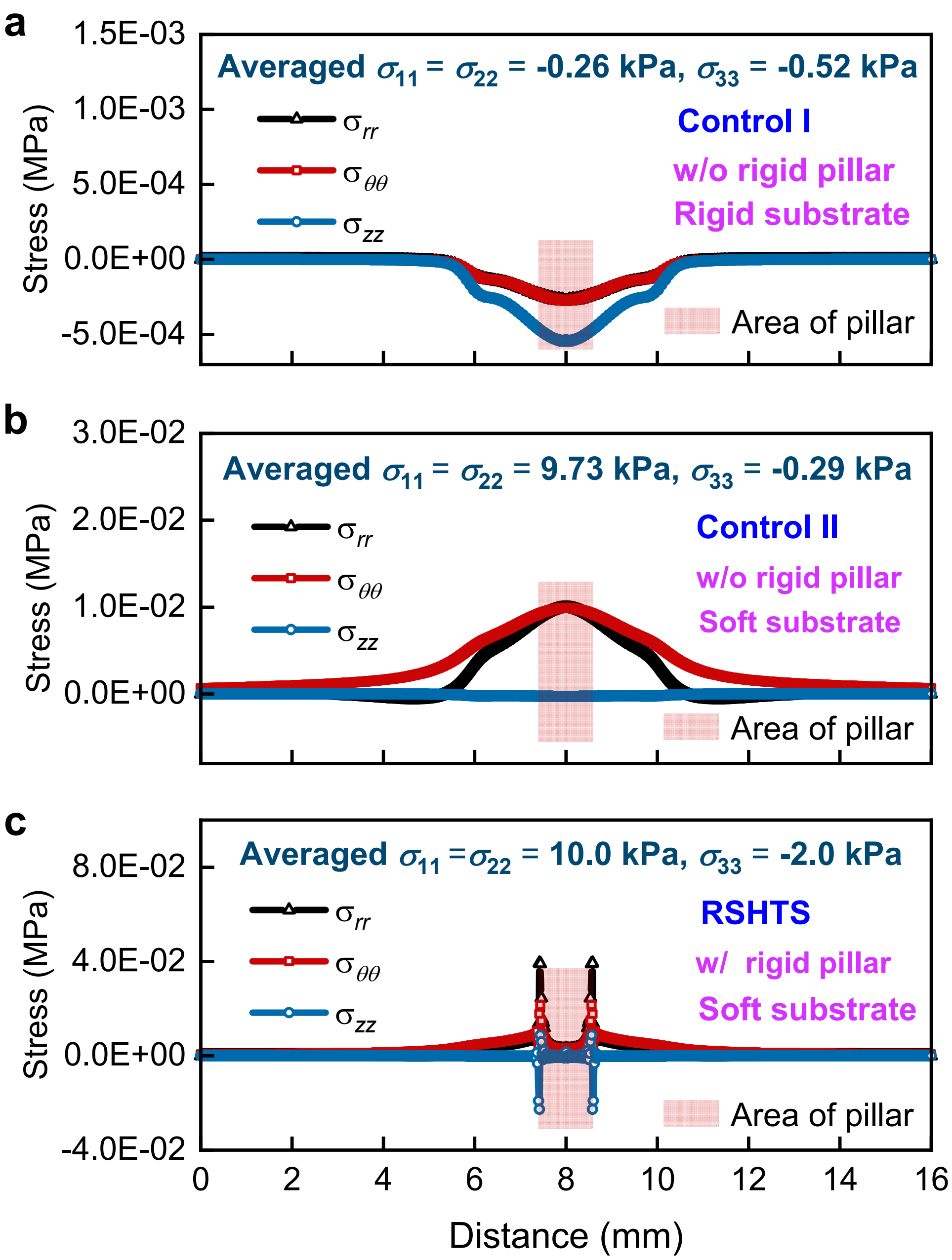

Supplementary Fig. 6 | Finite element simulation of stress σ along a path in cylindrical coordinate, and the averaged stress of sensory layer calculated by Eq. (10) for the area of pillar. **a** control sensor with rigid substrate (Control I). **b** Control sensor with soft substrate (Control II). **c** RSHTS. Note: cylindrical coordinate is adopted in the simulation, while for sensitivity evaluation using equation, averaged stress (σ_{11}, σ_{22}, σ_{33}) of piezoelectric layer beneath the pillar in cartesian coordinate is used.

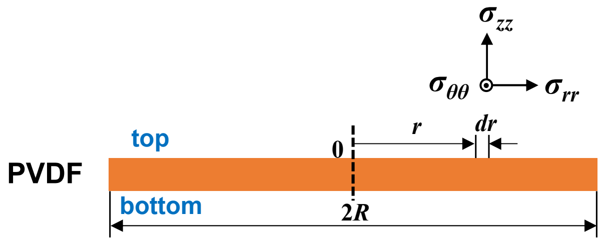

Supplementary Fig. 7 | Cross-sectional view of PVDF layer beneath the pillar.

The generated charge (Q) can be calculated by

$$Q = (d_{31}\sigma_{11} + d_{32}\sigma_{22} + d_{33}\sigma_{33})A$$
$$= d_{31}\pi\int_0^R (\sigma_{rr} + \sigma_{\theta\theta})r\,dr + d_{32}\pi\int_0^R (\sigma_{rr} + \sigma_{\theta\theta})r\,dr + d_{33}2\pi\int_0^R \sigma_{zz}\,r\,dr \quad (8)$$

where $A = \pi R^2$, R is the radius of electrode zone. σ_{rr}, $\sigma_{\theta\theta}$ and σ_{zz} is obtained from simulation results (Supplementary Figs. 6-7),

$$\begin{cases} \sigma_{rr} = \dfrac{\sigma_{rr\text{top}} + \sigma_{rr\text{bottom}}}{2} \\ \sigma_{\theta\theta} = \dfrac{\sigma_{\theta\theta\text{top}} + \sigma_{\theta\theta\text{bottom}}}{2} \\ \sigma_{zz} = \dfrac{\sigma_{zz\text{top}} + \sigma_{zz\text{bottom}}}{2} \end{cases} \quad (9)$$

Thus, the averaged stress σ_{11}, σ_{22}, σ_{33} is

$$\begin{cases} \sigma_{11} = \dfrac{\int_0^R (\sigma_{rr} + \sigma_{\theta\theta})r\,dr}{R^2} \\ \sigma_{22} = \dfrac{\int_0^R (\sigma_{rr} + \sigma_{\theta\theta})r\,dr}{R^2} \\ \sigma_{33} = \dfrac{2\int_0^R \sigma_{zz}\,r\,dr}{R^2} \end{cases} \quad (10)$$

where R is the radius of electrode zone.

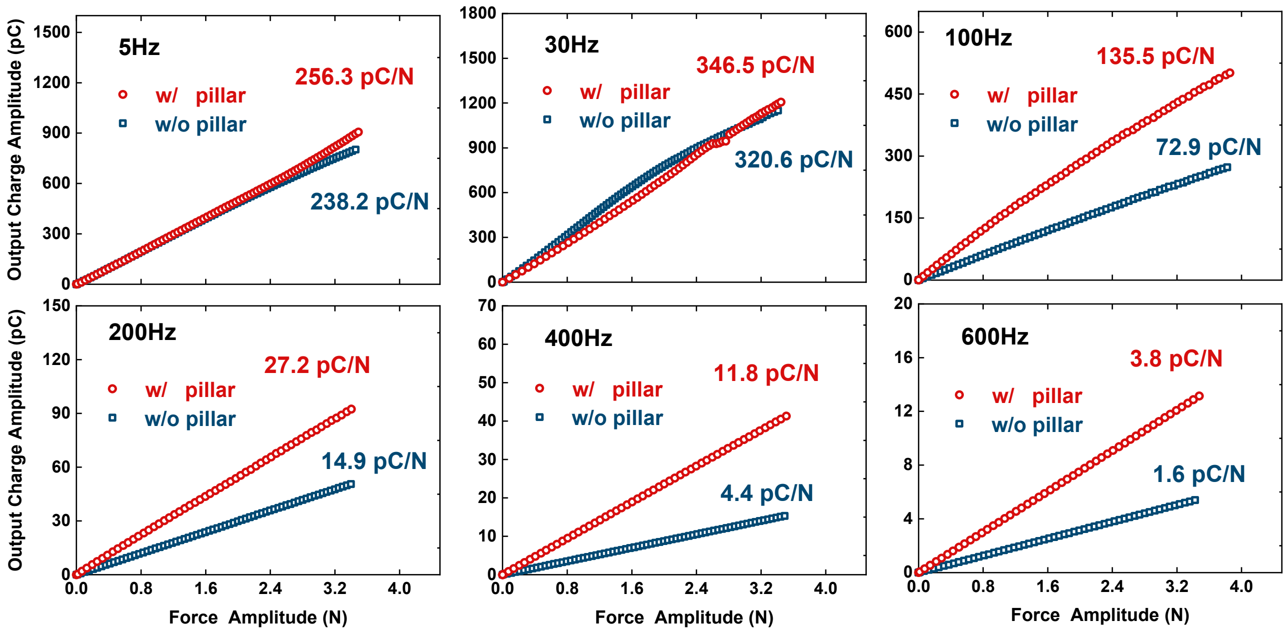

Supplementary Fig. 8 | Frequency response of the RSHTS and the control sensor without pillar (Control II). Output charge of RSHTS and control sensor as a function of applied normal force measured at 5–600 Hz. The experimental results show that the sensitivity of RSHTS with pillar is 1.1 @ 5 Hz, 1.1 @ 30 Hz, 1.9 @ 100 Hz, 1.8 @ 200 Hz, 2.7 @ 400 Hz, 2.4 @ 600 Hz times the sensitivity of control sensor without pillar.

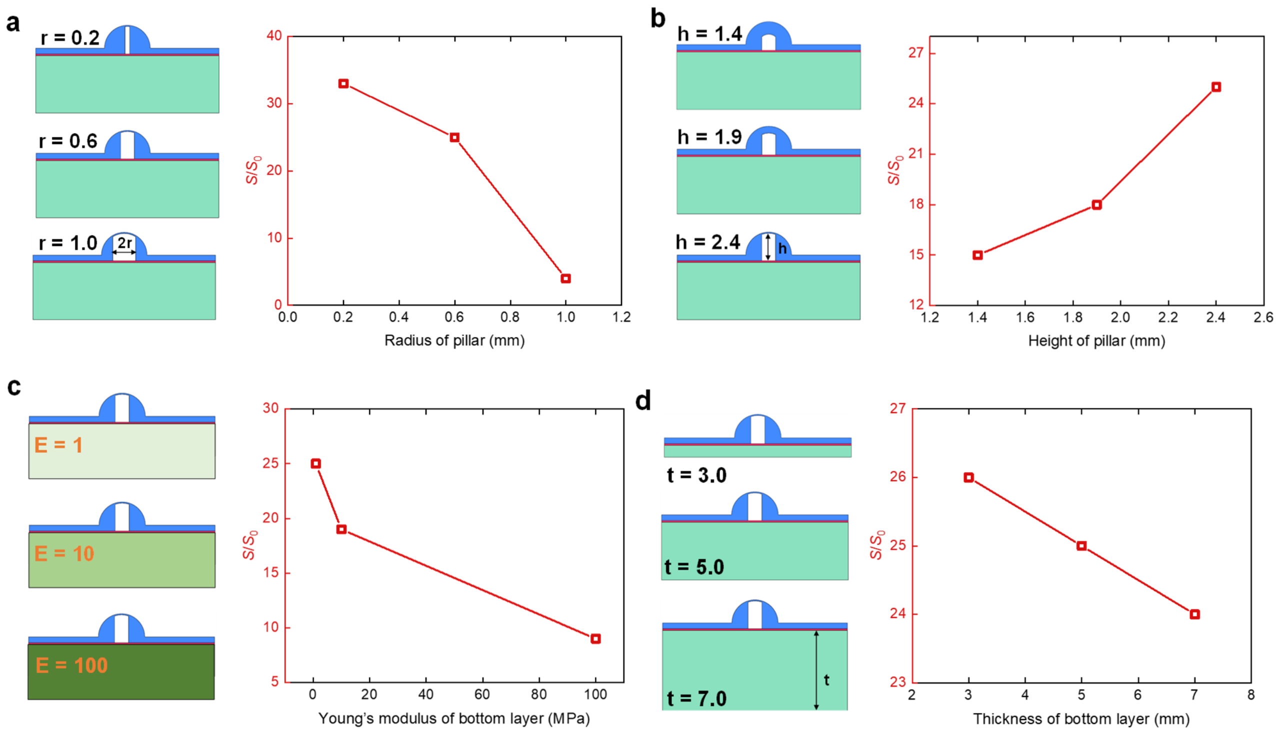

Supplementary Fig. 9 | Numerical simulation for sensitivity S/S_0 of the RSHTS (S normalised by the sensitivity S_0 of the conventional piezoelectric tactile sensor working in d_{33} mode) under different parameters, including pillar (a) radius and (b) height, (c) Young's modulus and (d) thickness of the bottom substrate.

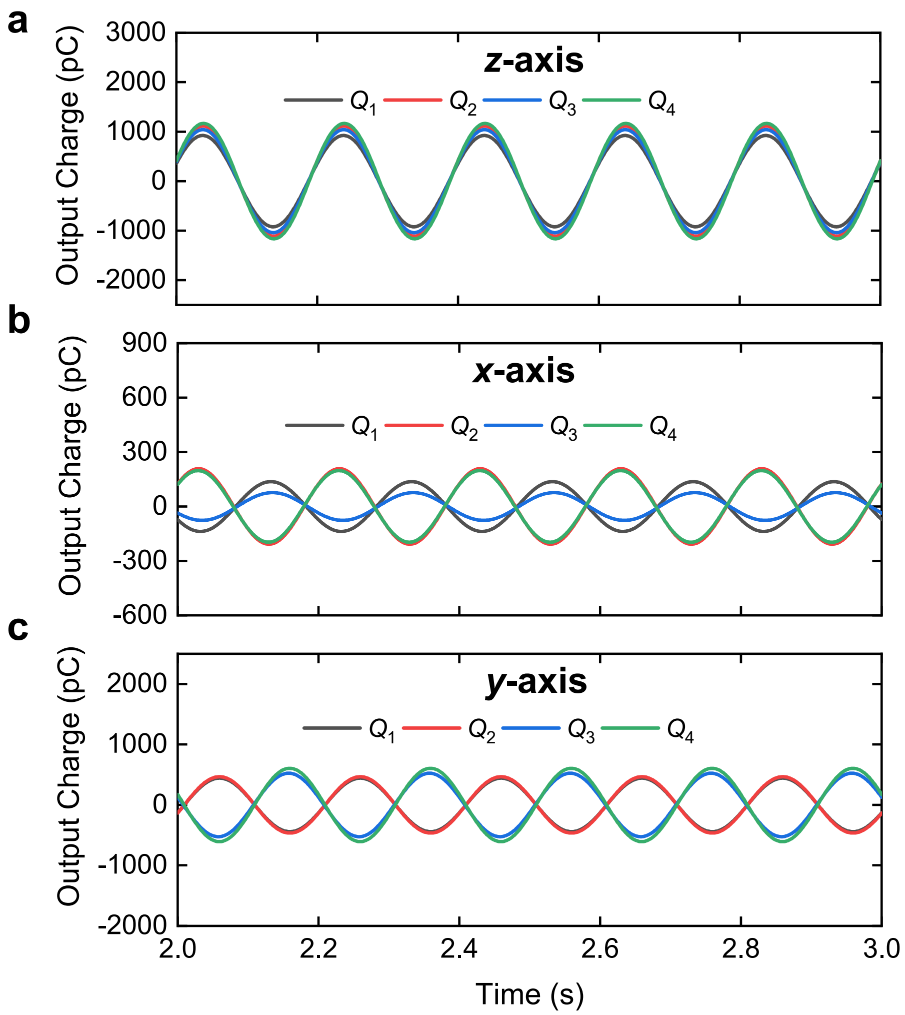

Supplementary Fig. 10 | Real-time output charges (Q_1, Q_2, Q_3, Q_4) of four piezoelectric capacitors (C_1, C_2, C_3, and C_4) under the sinusoidal forces (f = 5 Hz) in the x, y, and z axes. a Normal force in z-axis with an amplitude of 4.07 N. **b** Shear force in x-axis with an amplitude of 1.43 N. **c** Shear force in the y-axis with an amplitude of 1.44 N.

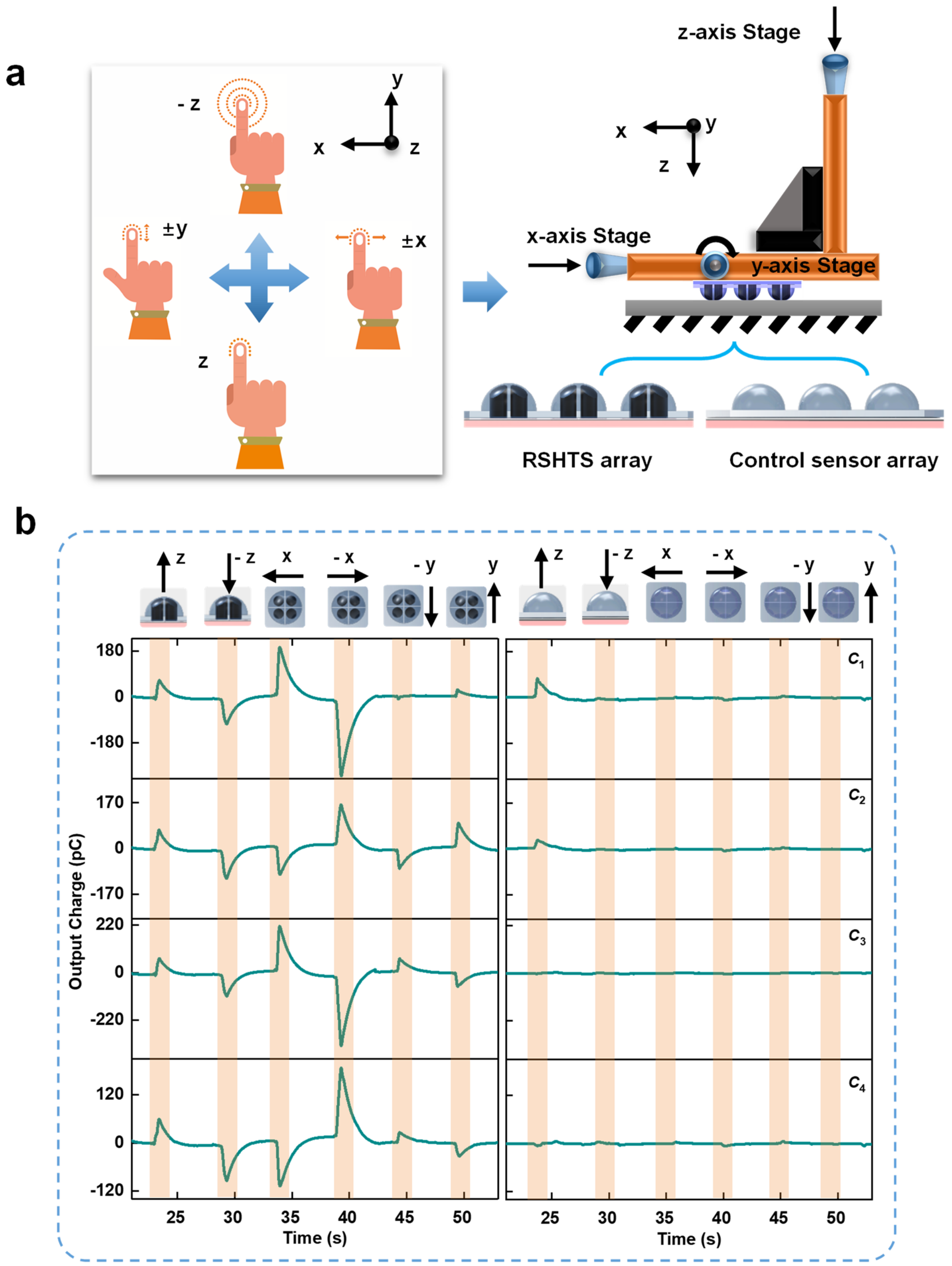

Supplementary Fig. 11 | Comparison of the signal direction detection between the RSHTS and the control sensor (without rigid pillars). a Schematic of the finger's six slipping directions. The sensor array is attached on a 3D micro stage to simulate the finger slipping by controlling the x, y, and z axes. **b** Taking one sensory unit as an example, four piezoelectric capacitor output charges of the RSHTS (left) and the control sensor (right) are recorded in real time, respectively.

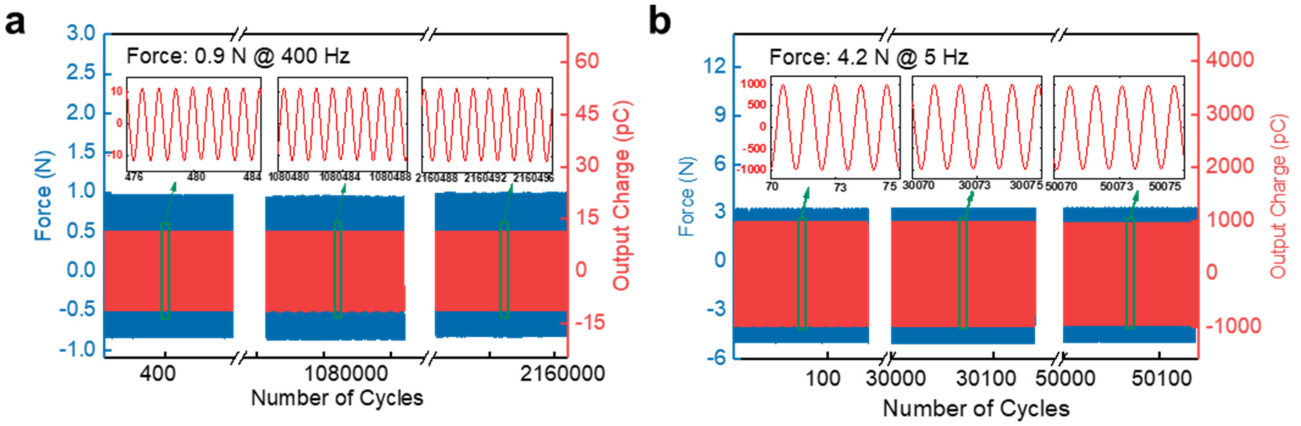

Supplementary Fig. 12 | Cycling stability of the RSHTS. a Signal stability over 2160000 cycles under a sinusoidal force of 0.9 N and 400 Hz. The three pictures on the top show the details of 8 cycles (from the 476th to the 484th cycle, the 1080480th to the 1080488th cycle and the 2160488th to the 2160496th cycle, respectively). **b** Signal stability over 50000 cycles under a sinusoidal force of 4.2 N and 5 Hz. The three pictures on the top show the details of 5 cycles (from the 70th to the 75th cycle, the 30070th to the 30075th cycle and the 50070th to the 50075th cycle, respectively).

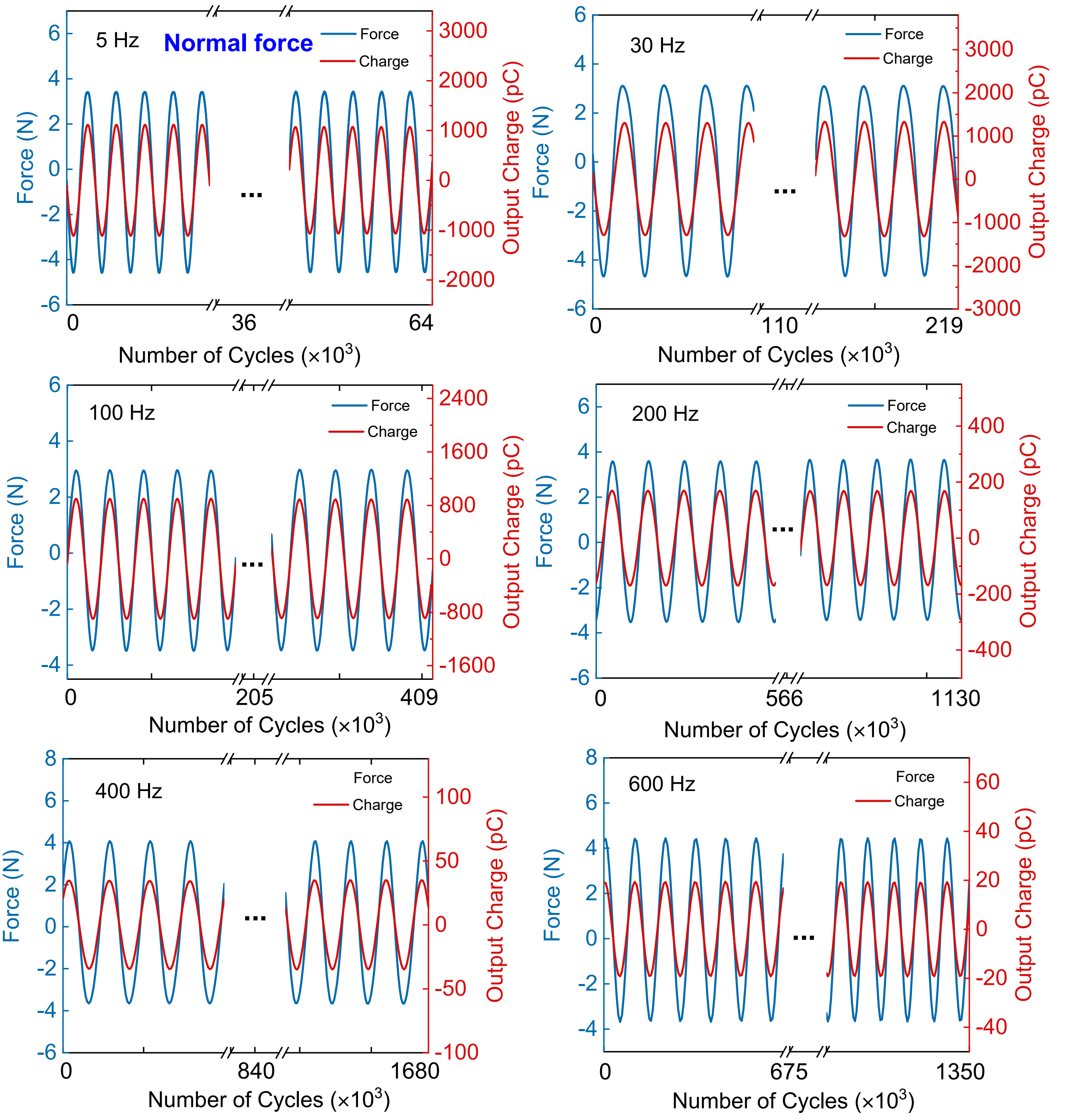

Supplementary Fig. 13 | Cycling stability of the RSHTS under a sinusoidal normal force range of 5–600 Hz.

Supplementary Table 3 The change of output charge of the RSHTS under normal force with different excitation frequencies based on Supplementary Fig. 13.

Frequency (Hz)	5	30	100	200	400	600
**Change of output charge (%)**	3.88	2.93	0.28	0.65	1.18	0.03

* The change of output charge is calculated by the average amplitude (10 cycles) at the start and end range of the recorded data during the experiment.

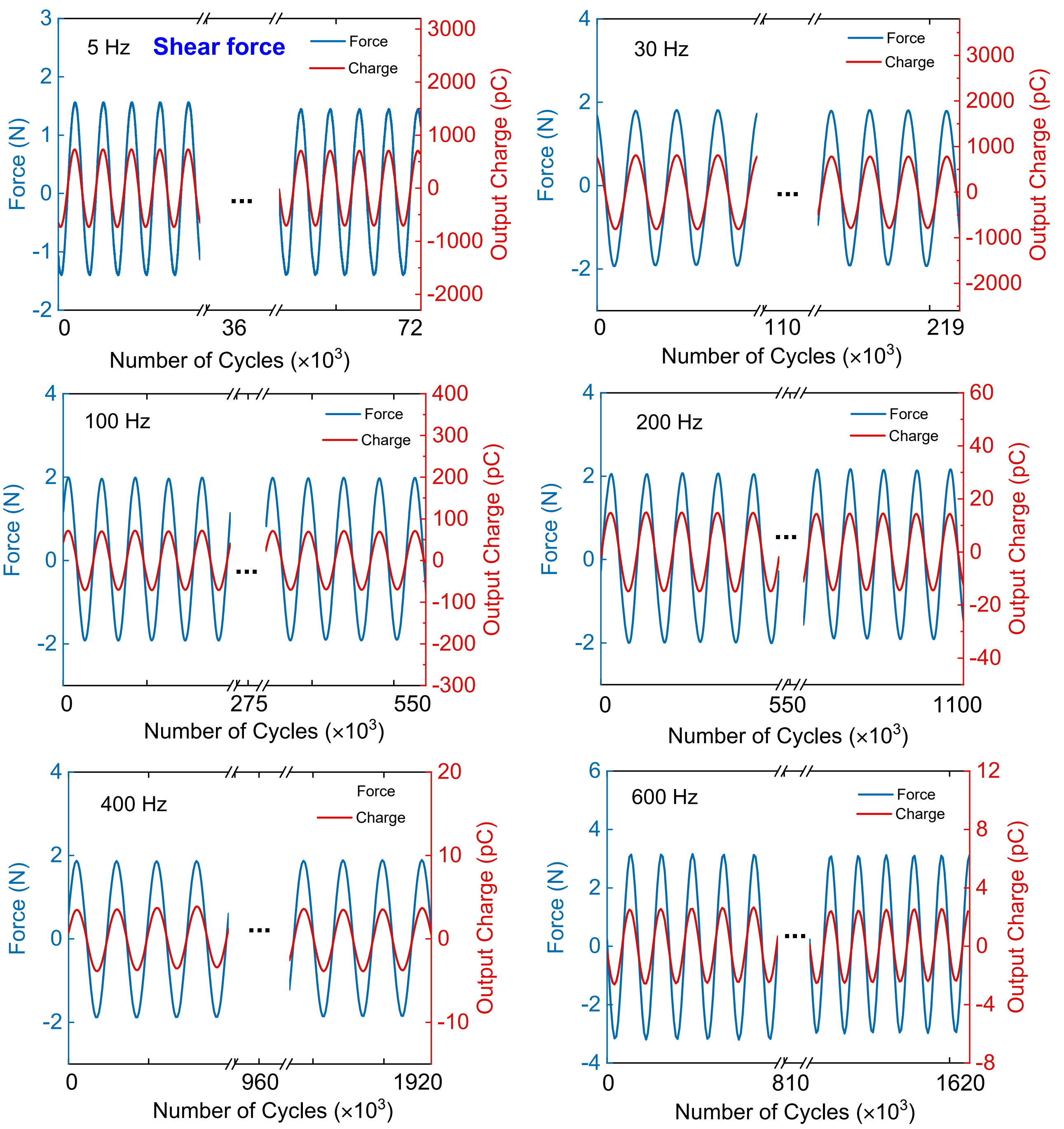

Supplementary Fig. 14 | Cycling stability of the RSHTS under a sinusoidal shear force range of 5–600 Hz.

Supplementary Table 4 The change of output charge of the RSHTS under shear force with different excitation frequencies based on Supplementary Fig. 14.

Frequency (Hz)	5	30	100	200	400	600
Change of output charge (%)	3.52	3.50	0.84	1.87	0.87	2.98

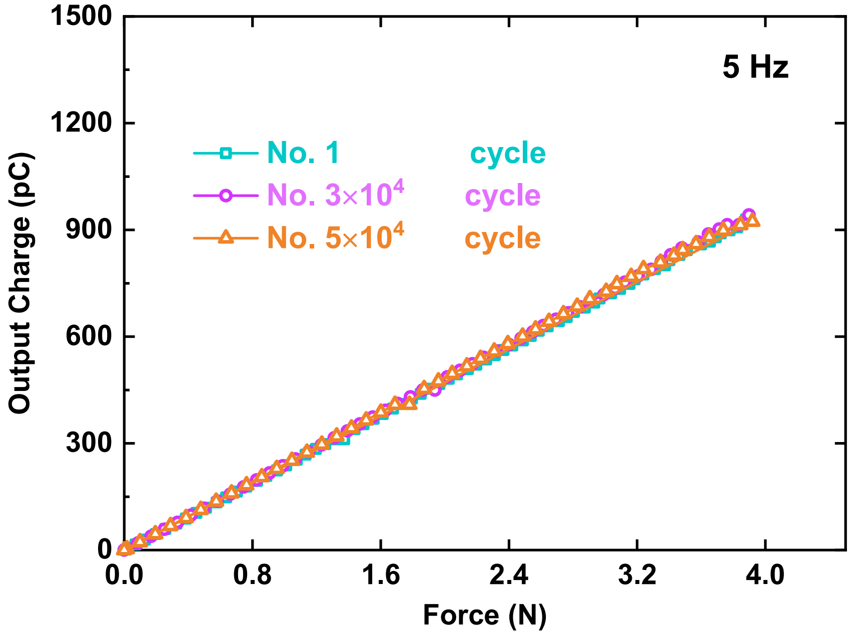

Supplementary Fig. 15 | Repeat performance of the RSHTS. Output charge of the RSHTS as a function of applied normal force measured at 5 Hz is repeated after 1, 3×10^4 and 5×10^4 cycles, and the sensitivity is correspondingly presented as 239.28, 241.96 and 241.18 pC N^{-1}.

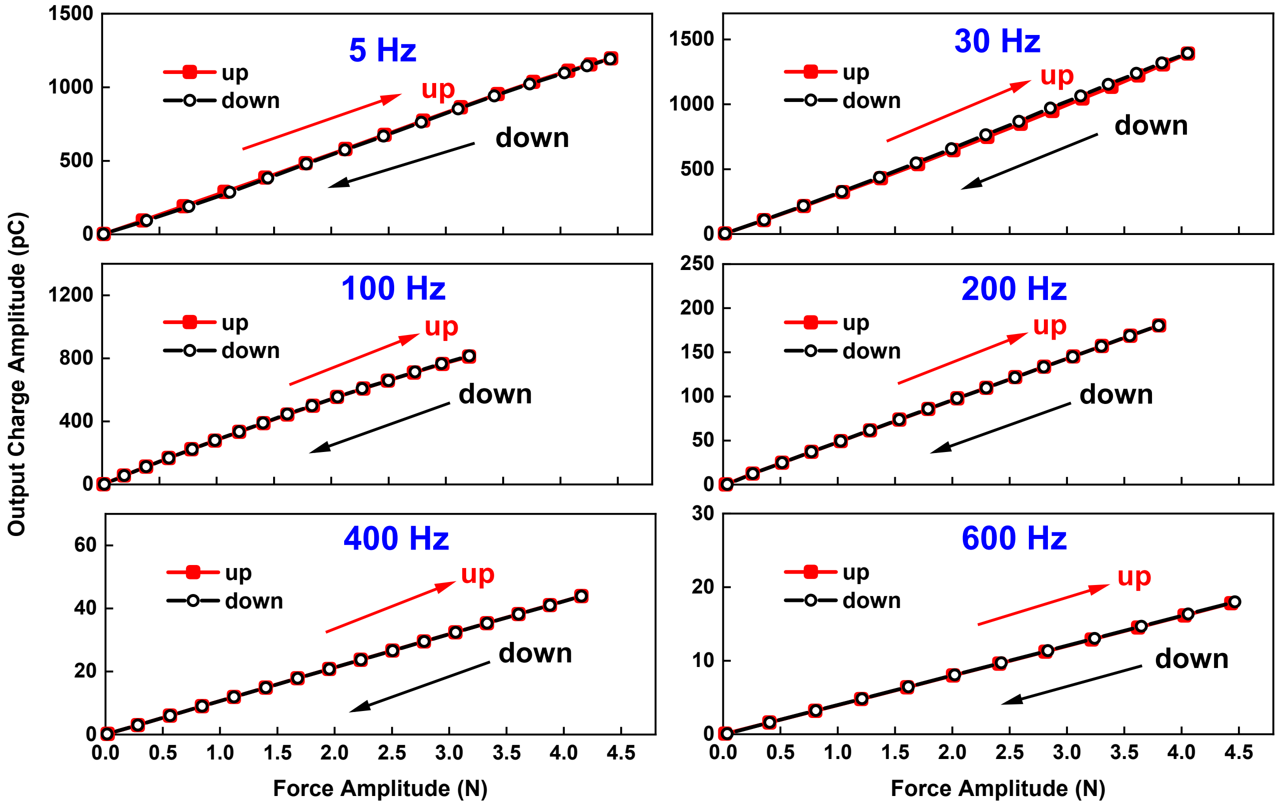

Supplementary Fig. 16 | Hysteresis performance of the RSHTS under normal force with excitation frequencies ranging from 5–600 Hz.

Supplementary Table 5 Hysteresis error of the RSHTS under normal force with excitation frequencies ranging from 5–600 Hz based on Supplementary Fig. 16.

Sensor	Frequency (Hz)	5	30	100	200	400	600
RSHTS	Hysteresis error (%)	0.30	1.61	0.51	0.13	0.41	0.68

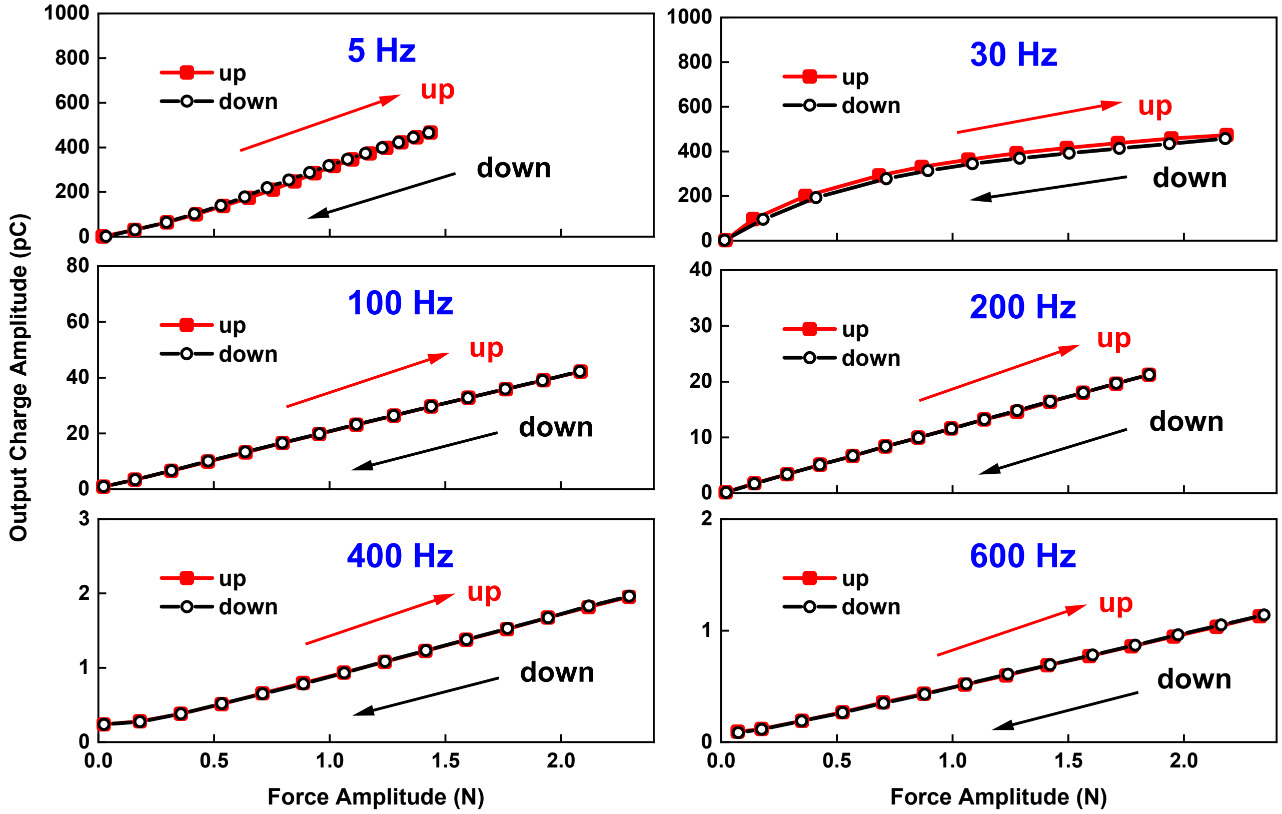

Supplementary Fig. 17 | Hysteresis performance of the RSHTS under shear force with excitation frequencies ranging from 5–600 Hz.

Supplementary Table 6 Hysteresis error of the RSHTS under shear force with excitation frequencies ranging from 5–600 Hz based on Supplementary Fig. 17.

Sensor	Frequency (Hz)	5	30	100	200	400	600
RSHTS	Hysteresis error (%)	1.88	5.13	0.36	1.13	0.45	1.45

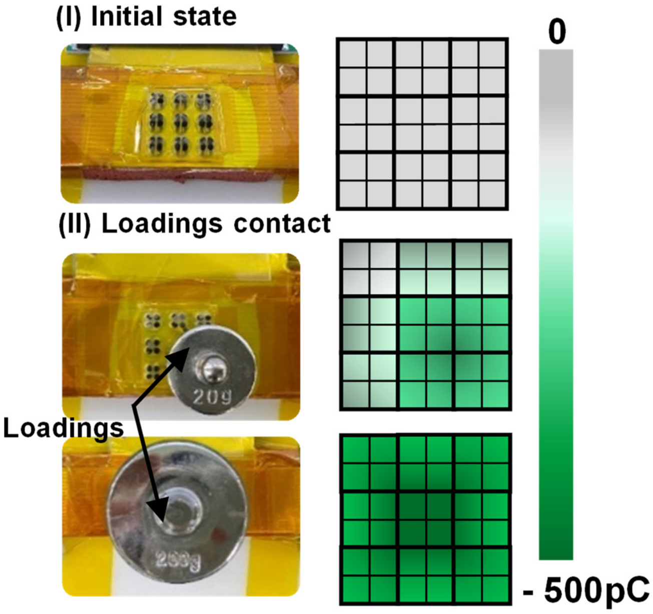

Supplementary Fig. 18 | Responding mapping of a 3 × 3 RSHTS array with different loadings (0 g, 20 g, 200 g).

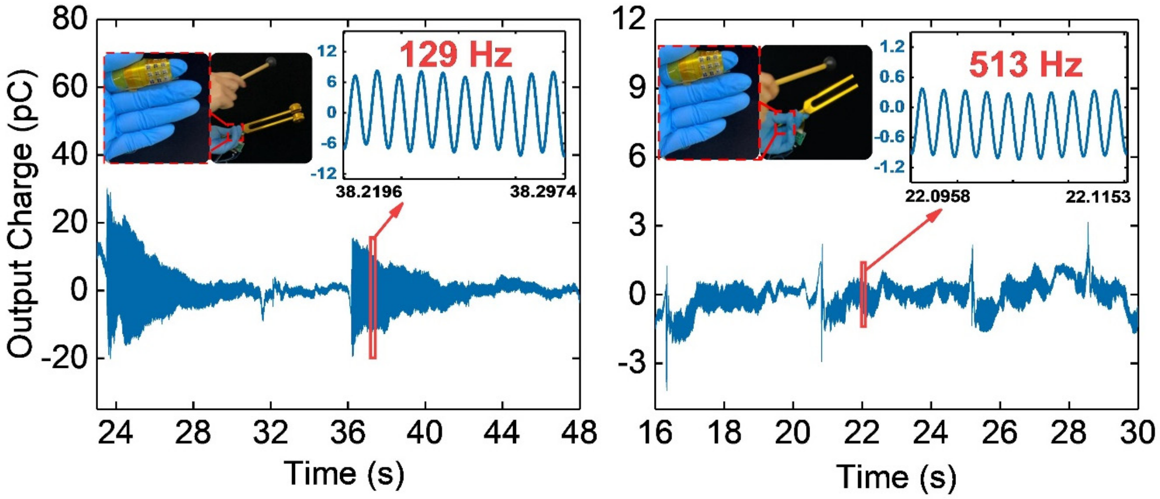

Supplementary Fig. 19 | High-frequency waveforms generated by tuning forks are recorded by RSHTS attached to human finger.

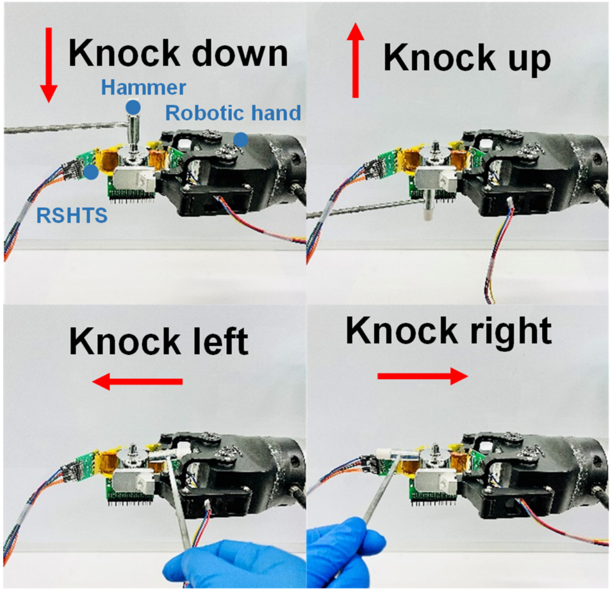

Supplementary Fig. 20 | A RSHTS-based robotic hand holding a mass block knocked by a hammer to simulate the scenario of a robot moving a big object and encountering a collision.

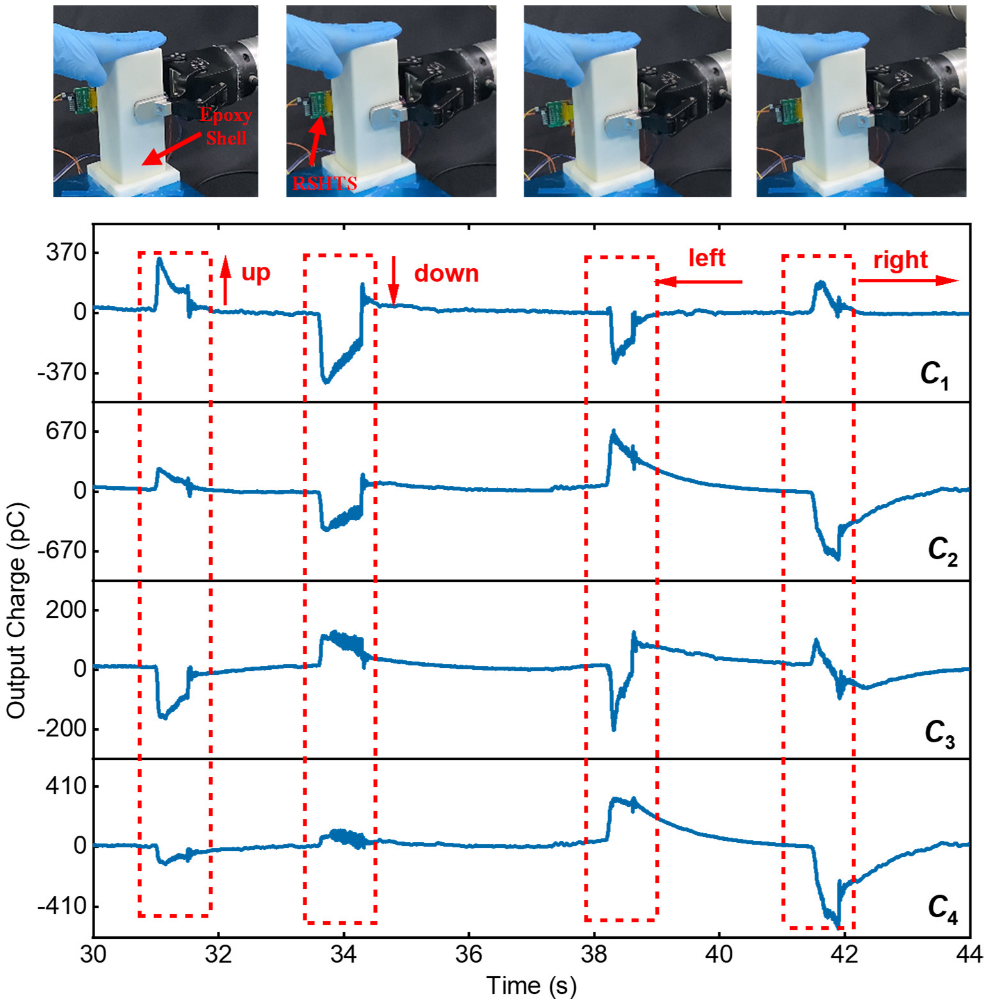

Supplementary Fig. 21 | Real-time output charges of four piezoelectric capacitors (C_1, C_2, C_3, and C_4) as the RSHTS installed on a robotic arm touching an epoxy shell to identify slipping directions.

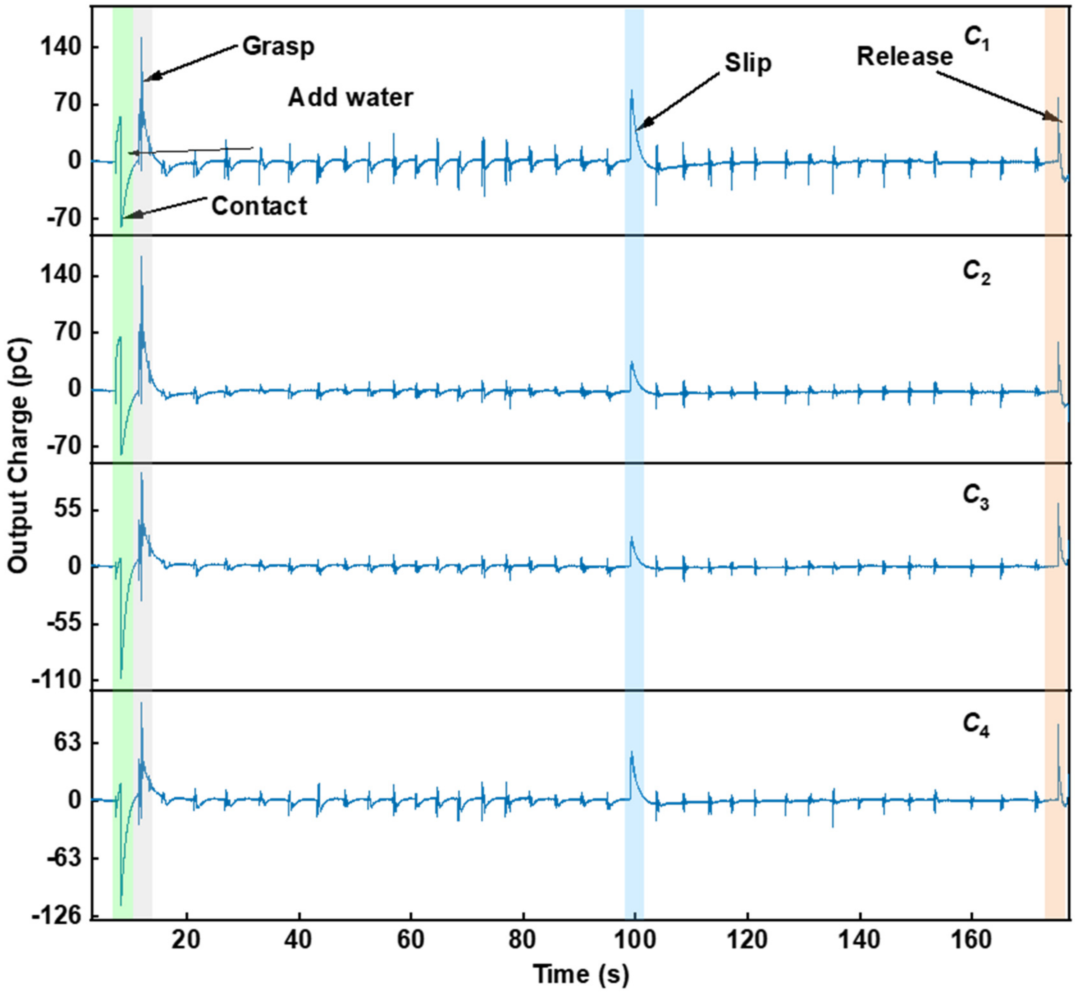

Supplementary Fig. 22 | Real-time output charge of four piezoelectric capacitors (C_1, C_2, C_3, C_4). From the four waveforms, the motions of the robotic hand contacting bottle, grasping bottle, receiving water, and releasing bottle can be identified.

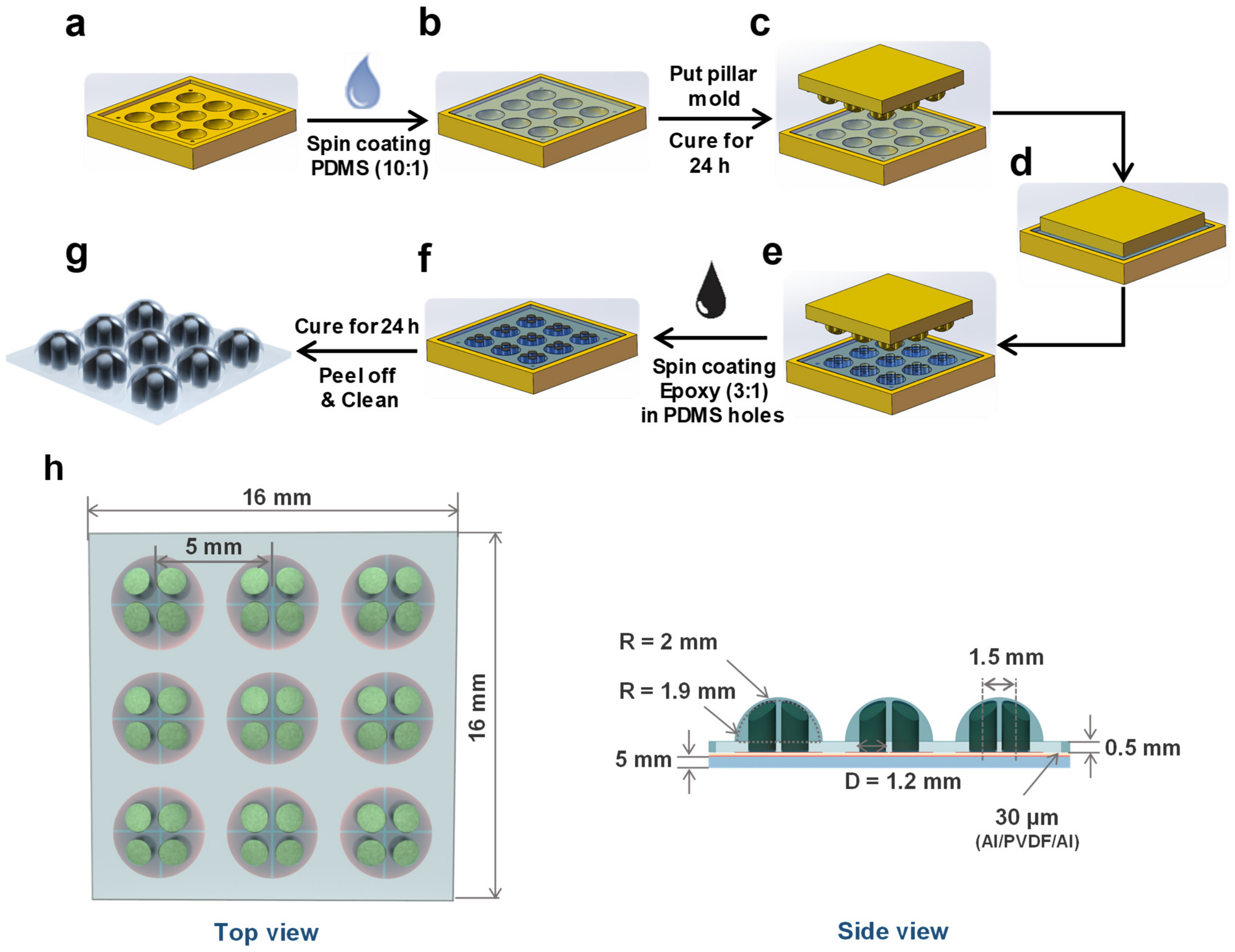

Supplementary Fig. 23 | Schematic of the fabrication process of rigid-soft hybrid force-transmission-layer. **a** 3 × 3 pit mold. **b** Pit mold with dome-shaped PDMS. **c** Pit mold and pillar mold. **d** Combining pit mold with pillar mold. **e** Separating pit mold and pillar mold for dome-shaped PDMS with holes. **f** Dome-shaped PDMS with rigid pillars in pit mold. **g** Rigid-soft hybrid top layer. Details can be found in the *Methods* section. **h** Top view and side view of RSHTS array.

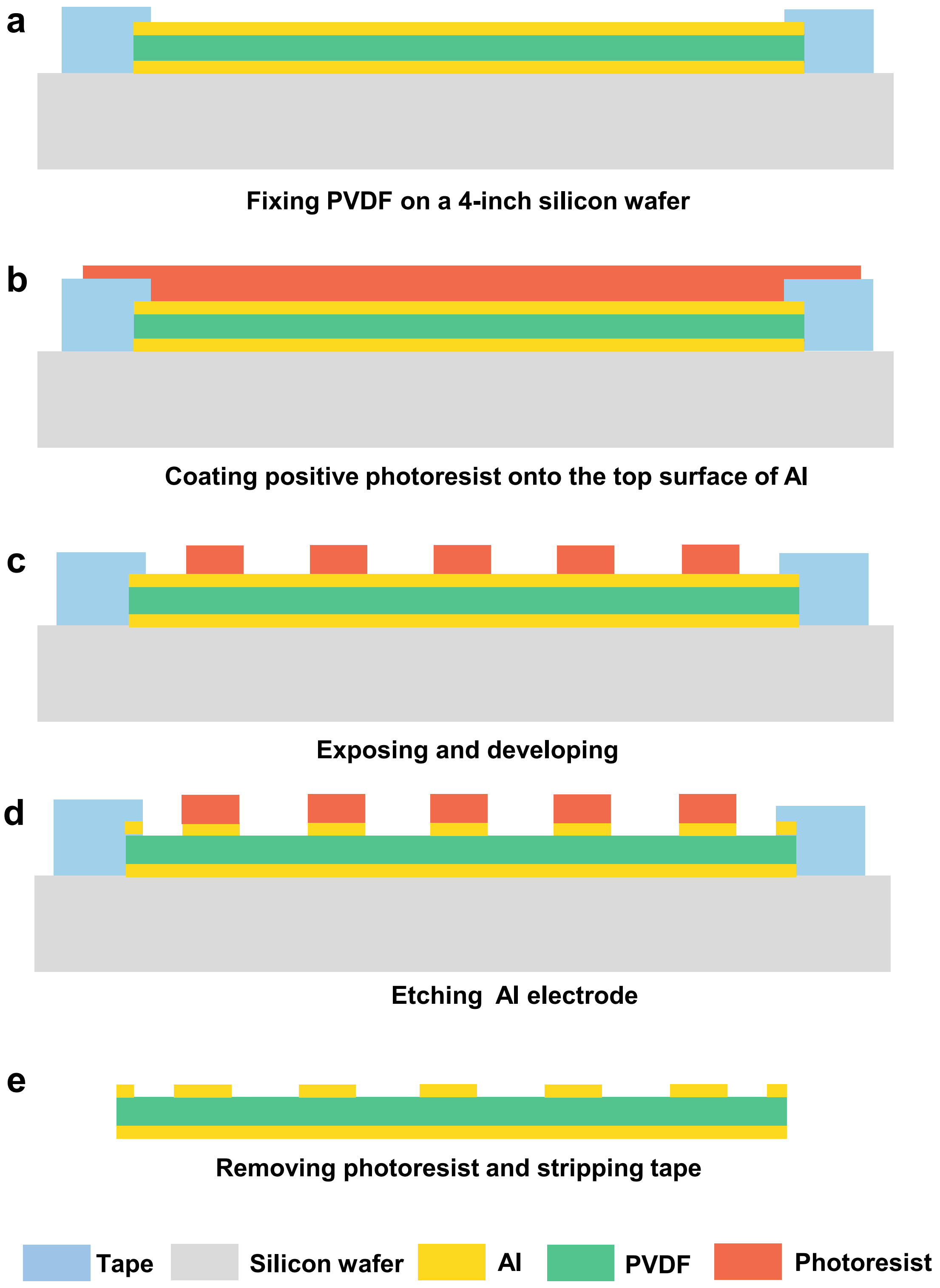

Supplementary Fig. 24 | Schematic of fabrication process of patterned PVDF film. Details can be found in the *Methods* section.

Supplementary References

1. Kolesar, E. S., Member, S. & Dyson, C. S. Object imaging with a piezoelectric robotic tactile sensor. *J. Microelectromechanical Syst.* **4**, 87–96 (1995).
2. Chuang, C. H. Flexible piezoelectric tactile sensors with structural electrodes array. *Lect. Notes Electr. Eng.* **49 LNEE**, 189–202 (2009).
3. Murat Koç, I. & Akça, E. Design of a piezoelectric based tactile sensor with bio-inspired micro/nano-pillars. *Tribol. Int.* **59**, 321–331 (2013).
4. Deng, W. *et al.* Cowpea-structured PVDF/ZnO nanofibers based flexible self-powered piezoelectric bendingmotion sensor towards remote control of gestures. *Nano Energy* **55**, 516–525 (2019).
5. Kim, M. S., Ahn, H. R., Lee, S., Kim, C. & Kim, Y. J. A dome-shaped piezoelectric tactile sensor arrays fabricated by an air inflation technique. *Sensors Actuators, A Phys.* **212**, 151–158 (2014).
6. Seminara, L. *et al.* Piezoelectric polymer transducer arrays for flexible tactile sensors. *IEEE Sens. J.* **13**, 4022–4029 (2013).
7. Yu, P., Liu, W., Gu, C., Cheng, X. & Fu, X. Flexible piezoelectric tactile sensor array for dynamic three-axis force measurement. *Sensors (Switzerland)* **16**, 1–15 (2016).
8. Kärki, S., Lekkala, J., Kuokkanen, H. & Halttunen, J. Development of a piezoelectric polymer film sensor for plantar normal and shear stress measurements. *Sensors Actuators A Phys.* **154**, 57–64 (2009).
9. Dargahi, J. A piezoelectric tactile sensor with three sensing elements for robotic, endoscopic and prosthetic applications. *Sensors Actuators A Phys.* **80**, 23–30 (2000).
10. Khan, S., Tinku, S., Lorenzelli, L. & Dahiya, R. S. Flexible tactile sensors using screen-printed P(VDF-TrFE) and MWCNT/PDMS composites. *IEEE Sens. J.* **15**, 3146–3155 (2015).
11. Zhu, P. *et al.* Flexible 3D Architectured Piezo/Thermoelectric Bimodal Tactile Sensor Array for E-Skin Application. *Adv. Energy Mater.* **10**, 1–8 (2020).
12. Chen, X. *et al.* Scalable imprinting of flexible multiplexed sensor arrays with distributed piezoelectricity-enhanced micropillars for dynamic tactile sensing. *Adv. Mater. Technol.* **5**, 1–9 (2020).
13. Li, C. *et al.* Flexible dome and bump shape piezoelectric tactile sensors using PVDF-TrFE copolymer. *J. Microelectromechanical Syst.* **17**, 334–341 (2008).
14. Cheng, X., Gong, Y., Liu, Y., Wu, Z. & Hu, X. Flexible tactile sensors for dynamic triaxial force measurement based on piezoelectric elastomer. *Smart Mater. Struct.* **29**, 075007 (2020).
15. Liu, Z. H., Pan, C. T., Lin, L. W. & Lai, H. W. Piezoelectric properties of PVDF/MWCNT nanofiber using near-field electrospinning. *Sensors Actuators A Phys.* **193**, 13–24 (2013).
16. Yu, K., Yoon, M., Kwon, T. & Lee, S. Distributed flexible tactile sensor system. *Int. J. Appl. Electromagn. Mech.* **18**, 53–65 (2003).
17. Maita, F. *et al.* Ultraflexible Tactile Piezoelectric Sensor Based on Low-Temperature Polycrystalline Silicon Thin-Film Transistor Technology. *IEEE Sens. J.* **15**, 3819–3826 (2015).
