# Supplementary material for: Finger-inspired rigid-soft hybrid tactile sensor with superior sensitivity at high frequency
Source: Nat Commun. 2022 Aug 29;13:5076. doi: 10.1038/s41467-022-32827-7 (PMC9422944; doi:10.1038/s41467-022-32827-7)
Supplement: Supplementary file 3 — Description of Additional Supplementary File [file 41467_2022_32827_MOESM3_ESM.docx]

**File Name:** Supplementary Video 1
**Description:** A 3 × 3 RSHTS array is mounted on the human finger for simulating the tactile system of robots. Three tuning forks with different natural frequencies are held by the human hand, and the output charge of RSHTS after multiple shock excitations is recorded. The recorded real-time curves show that RSHTS can recognize vibration frequency (129, 257 and 513 Hz) and change process of the external dynamic signal.

**File Name:** Supplementary Video 2
**Description:** The RSHTS array can be used to detect collision force directions. The RSHTS-based robotic hand is used to hold a mass block. The block is knocked by a hammer from four different directions to simulate the robot’s moving object and encountering a collision. Taking one sensory unit as an example, the real-time output of four piezoelectric capacitors is recorded, from which the knock directions can be determined by monitoring the output charge polarity.

**File Name:** Supplementary Video 3
**Description:** The RSHTS array is attached to the robotic hand for monitoring the water-pouring process: a bottle is initially placed on a platform, then the robotic hand contacts and grasps the bottle, and water are added drop by drop, finally, the bottle is released. The real-time output charge of one piezoelectric capacitor of the RSHTS is recorded. From the real-time curve, it can be seen that the whole dynamic process of the robotic hand catching bottle, grasping bottle, receiving water and releasing bottle can be clearly identified.
